# Genome-wide association study of serum tumor markers in Southern Chinese Han population

**DOI:** 10.1186/s12885-022-09236-6

**Published:** 2022-02-10

**Authors:** Xiukuan Li, Fenghua Bai, Xingwei Wei, Tianbo Jin, Chen Li, Yutian Zhang, Mei Lin, Xiaoli Zhou, Yufei Xie, Chanyi He, Qi Lin, Ping He, Shuyuan Chu, Yipeng Ding

**Affiliations:** 1 Department of General Practice, Hainan General Hospital, #19, Xiuhua Road, Xiuying District, Haikou, Hainan 570311 P.R. China; 2 Department of Outpatient, Meitai Health Center, Lingao County, Haikou, Hainan 571824 P.R. China; 3 Department of Science and Education, Hainan General Hospital, Haikou, Hainan 570311 P.R. China; 4 Key Laboratory of Molecular Mechanism and Intervention Research for Plateau Diseases of Tibet Autonomous Region, School of Medicine, Xizang Minzu University, Xianyang, 712082 Shaanxi China; 5 Key Laboratory of Resource Biology and Biotechnology in Western China (Northwest University), Ministry of Education, School of Life Sciences, Northwest University, Xi’an, 710069 Shaanxi China; 6 Xi’an 21st Century Biological Sicence and Technology Co., Ltd, Xi’an, 712000 Shaanxi China; 7 Laboratory of Respiratory Disease, Affiliated Hospital of Guilin Medical University, #15 Lequn Road, Guilin City, Guangxi Zhuang Autonomous Region, Guilin, 541001 Guangxi China

**Keywords:** Tumor markers, GWAS, Imputation, Southern Chinese Han population

## Abstract

**Background:**

Serum indicators AFP, CA50, CA125, CA153, CA19-9, CEA, f-PSA, SCC-Ag have been confirmed as tumor markers (TMs). We conducted a genome-wide association study on 8 tumor markers of our 427 Han population in southern China, in order to identify genetic loci that are significantly associated with the level of 8 tumor markers.

**Methods:**

We use Gene Titan multi-channel instrument and Axiom Analysis Suite 6.0 software for genotyping. We used IMPUTE2 software for imputation, and 1000 Genomes Project (Phase 3) was used as haplotype reference. After necessary quality control and statistical analysis, genetic loci genome-wide associated with TMs (*p* < 5E-8) will be identified. Finally, we selected Top SNPs (*p* < 5E-7) from the GWAS results for replication test. We used SPSS software to draw the distribution box plots of serum TMs under different genotypes of significant loci.

**Results:**

The results showed that there were only *MUC1* (mucin 1)-rs4072037 significantly genome-wide associated with CA153 (*p* = 1.28E-18). However, we found that a total of 30 genetic loci have a suggestively significant genome-wide association with the level of 8 serum tumor markers (*p* < 5E-6). Then 3 Top SNPs (*p* < 5E-7) were selected for replication verification. The results showed that *MUC1*-rs4072037 was still significantly associated with CA153 in another population (*p* = 3.73E-08). Comparing with the TT genotype of rs4072037, the CA153 level was higher under CC or CT genotype of rs4072037.

**Conclusion:**

*MUC1*-rs4072037 is significantly genome-wide associated with CA153 level. There are 30 genetic loci suggestively genome-wide associated with level of tumor markers among the Han population from Southern China.

**Supplementary Information:**

The online version contains supplementary material available at 10.1186/s12885-022-09236-6.

## Background

Tumor marker (TM) is a series of substances that reflect the existence or development of tumors. It not only plays a role in the disease detection and target treatment of tumor patients, but also has important practical value in the anti-cancer screening of healthy people. Numerous studies have confirmed that the levels of certain related indicators in serum are closely associated with the occurrence and development of one or more tumors, and can be regarded as tumor markers. Such as alpha fetoprotein (AFP): liver cancer [1, 2]; CA19-9: pancreatic cancer; gastric cancer; colorectal cancer, etc. [3–6]; CA125: ovarian cancer [7]; CA153: breast cancer [8]; CA50: pancreatic cancer [9]; carcino embryonic antigen (CEA): colorectal cancer [10]; free Prostate Specific Antigen (f-PSA): prostate cancer [11]; squamous cell carcinoma antigen (SCC-Ag): squamous cell carcinomas [12], etc. In addition, previous studies suggested that the level of tumor markers such as CA19-9, CEA and AFP may differ between healthy individuals and cancer patients due to individual genetic differences and environmental factors [13–17]. Therefore, the detection of these tumor marker levels is helpful to monitor the occurrence and development of specific tumors in specific clinical populations.

Genome-wide association study (GWAS) has become the main common method for studying complex diseases and their susceptibility genes because of its ability to cover single nucleotide polymorphisms in the whole genome [18]. It can more effectively find genetic loci associated with the occurrence and development of diseases. GWAS can perform population-level statistical analysis of genotype and phenotype, so as to helping us identify genetic loci related to phenotypic changes. Up to now, numerous SNPs associated with levels of tumor makers among different populations have been reported [13, 17, 19–21]. SNPs significantly associated with levels of tumor markers in specific populations can be identified through GWAS. However, there are few studies few studies on tumor markers GWAS. He, M. et al. identified several loci associated with CA19-9, CEA and AFP levels through GWAS studies among 3451 healthy participants [13]. The above reminds us that it is of great significance to identify genetic loci associated with tumor marker levels through GWAS, which provides new ideas and data supplement for further understanding the genetic mechanism of serum TMs in specific populations.

Therefore, we conducted a genome-wide association study on 8 serum tumor markers (AFP, CA125, CA19-9, CA153, CA50, CEA, f-PSA, SCC-Ag) in the Han population from southern China. With a view to discovering genetic loci that are significantly associated with serum tumor markers in the Han population from southern China. Our study will provide valuable reference for clinical monitoring and diagnosis of the occurrence and development of tumors among Han population from southern China.

## Materials and method

### Study subjects and DNA extraction

The subjects consisted of 427 healthy individuals (221 males and 206 females) from health examination center. The inclusion criteria for all participants included: age mainly 40-50 years old, no family history of tumor, no disease (at least within 2 weeks), and no medication (at least within 2 weeks). Then the whole bloods of the participants were collected, and we extracted the whole genome DNA. The specific operation procedure was carried out according to the kit instructions (GoldMag, Xi’an). Subsequently, a GWAS study was conducted on 8 serum tumor markers in the participating population, which are AFP, CA50, CA125, CA153, CA19-9, CEA, f-PSA, SCC-Ag.

### Genotyping and quality control

In our study, Thermo Fisher Genotyping Chip was used (Applied Biosystems™ Axiom™ Precision Medicine Diversity Array, PMDA). We use Gene Titan multi-channel instrument and Axiom Analysis Suite 6.0 software for genotyping. We performed a full gene scan through Axiom, and the results showed that within our sample range (427 participants), there were a total of 874,190 loci. After excluding Indel, copy number variation, sex chromosomes and duplicate sites, we then perform the necessary quality control on the remaining sites (sample call rate> 0.95, maker call rate> 0.90, HWE > 5 × 10^-6^). In the end, there are 796,288 loci left before we perform the imputation.

### Imputation and quality control

We used IMPUTE2 software and used the haplotype of the 1000 Genomes Project (Phase 3) as a reference for imputation. After imputation, there are many poor quality loci, which need to be filtered out. Loci need to be filtered out include loci with MAF=0, loci with MAF<0.01 and info>0.3, and loci with excessive deletion rates (98% or more). Finally, the loci that meet the following conditions will be retained. These conditions include: sample call rate > 95%, marker call rate > 90%, HWE > 5 × 10^-6^, Allele = 2. In the end, a total of 6423,076 SNPs were used for subsequent analysis.

### Statistical analysis

We use Gold Helix SNP & Variation Suite 8.7 version for association analysis. We mainly use the Mixed Linear Model-additive genetic model, and add the IBD matrix to the model to detect the SNPs associated with each indicator. All data in our study were adjusted by age and gender. At the same time, we also constructed Manhattan plots and Quantile-quantile plots related to serum TMs. The *p*-value is less than 5E-8, which means the genetic polymorphism is genome-wide significantly associated with the levels of tumor markers. Genetic polymorphism with *p*-value less than 5E-6 suggests that it may have a suggestively significant genome-wide association with tumor markers level [22].

### Repeat detection

SNPs with *p* < 5E-7 were selected as Top SNPs from all SNPs identified by GWAS, and then they were repeatedly verified among 398 participants from the health examination center of Hainan General Hospital (different from the participants in the GWAS analysis). We removed the extreme measurement values (mean > ± 3 sd) of each serum TMs, and then normalized them with rankbaseINTs. Genotyping was performed by Agena MassARRAY. Then, the association analysis between Top SNPs and serum TMs was analyzed by Gold Helix SNP & Variation Suite 8.7. Through the mixed linear model-additive genetic model, while adding the IBD matrix to the model, the correlation between each tumor marker and Top SNPs will be analyzed. The final results were all adjusted by age and gender. And we also use False Discovery Rate analysis to perform correction on positive results. Finally, SPSS software was used to draw the distribution box plots of serum TMs under different genotypes of significant loci. In this part, *p* < 0.05 indicates that the candidate SNPs are significant associated with the 8 serum TMs.

## Result

### GWAS identified 31 single nucleotide polymorphisms

A total of 427 Southern Chinese Han people participated in the GWAS assessment of this study. The number of participants, and ‘mean ± standard’ of each serum TMs were summarized in Table 1. There were differences in sample sizes for each indicator, which was due to data missing during sample collection.

**Table 1:** Sample information divided by different tumor markers

Tumor markers	Number^**a**^	Mean ± SD	Age (years) Mean ± SD	Gender	
				Male	Female
CEA	424	1.84 ± 0.91	44.29 ± 9.26	217 (51.18%)	207 (48.82%)
AFP	427	3.15 ± 1.35	44.29 ± 9.27	221 (51.76%)	206 (48.24%)
CA19-9	415	9.88 ± 5.34	44.30 ± 9.24	216 (52.05%)	199 (47.95%)
CA125	424	11.23 ± 4.97	44.23 ± 9.35	220 (51.89%)	204 (48.11%)
CA50	265	6.32 ± 3.30	43.54 ± 9.65	171 (64.53%)	94 (35.47%)
f-PSA	233	0.30 ± 0.17	43.24 ± 9.77	154 (66.09%)	79 (33.91%)
CA153	165	10.31 ± 4.96	45.77 ± 8.69	51 (30.91%)	114 (69.09%)
SCC	422	0.49 ± 0.11	44.33 ± 9.18	219 (51.90%)	203 (48.10%)

*N* Number, *AFP* Alpha-fetoprotein, *CA50* Carbohydrate Antigen CA-50, *CA125* Carbohydrate Antigen CA125, *CA153* Carbohydrate Antigen CA153, *CA19-9* Carbohydrate Antigen CA19-9, *CEA* Carcino embryonic Antigen, *f-PSA* free Prostate Specific Antigen, *SCC-Ag* Squamous Cell Carcinoma Antigen

^a^There were differences in sample sizes for each indicator, which was due to data missing during sample collection

**Fig. 1:**
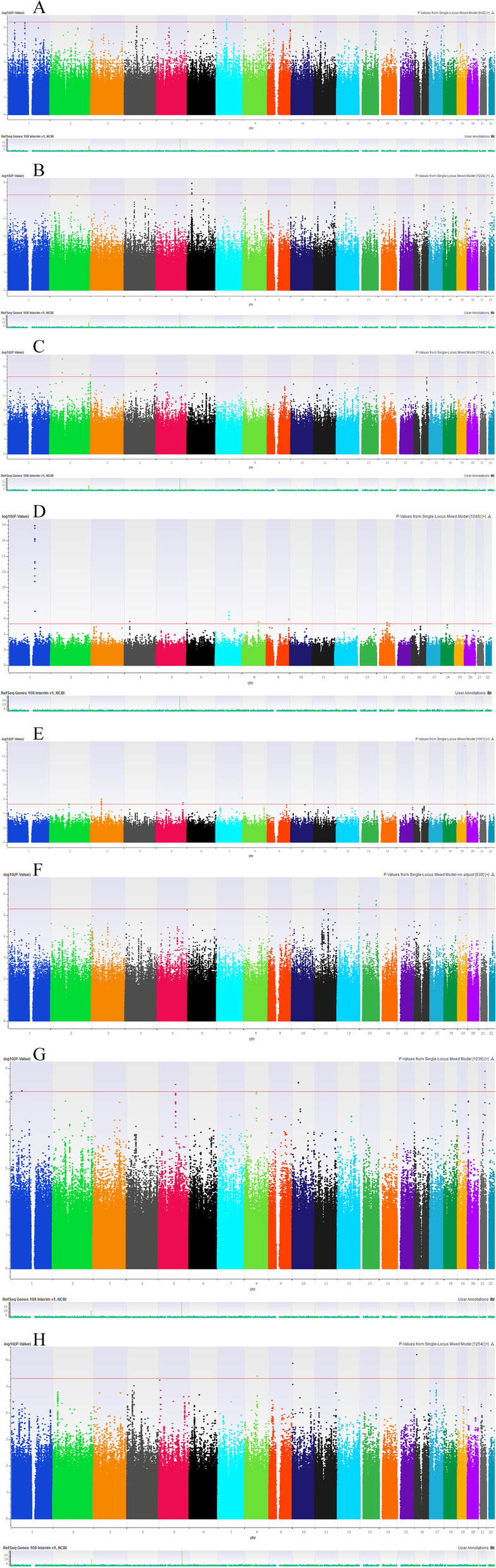
Manhattan graph of the results of the genome-wide association study. **A** AFP; **B** CA50; **C** CA125; **D** CA153; **E** CA19-9; **F** CEA; **G** f-PSA; **H** SCC-Ag. The chromosomes are displayed on the x-axis, while the y-axis represents the −log_10_ of the *p*-value. The red line represents the cut-off value of the suggestively genome-wide significance (5.0×10 ^-6^)

The results showed that there were only MUC1 (mucin 1)-rs4072037 significantly genome-wide associated with CA153 (*p* = 1.28E-18). However, we found that a total of 30 genetic loci have a suggestively significant genome-wide association with the level of 8 serum tumor markers. (*p* < 5E-6). Specifically, the genetic loci with suggestively genome-wide significances with 8 serum tumor markers are as follows: AFP: rs1524622 (*LOC100287704*/*LOC100287834*), rs143687479 (*SGCZ*), rs10425983 (*ZNF444*/*GALP*); CA50: rs112401204 (*RBFOX2*/*APOL3*), rs9295778 (*LINC00533*/*LINC01623*); CA125: rs75332508 (*GCFC2*/*LRRTM4*), rs12476806 (*LINC01790*/*SLC39A10*), rs7563672 (*D2HGDH*/*GAL3ST2*), rs6554915 (*LOC728613*/*MIR4277*), rs2028385 (*CDK17*/*CFAP54*); CA153: rs112804057 (*PCDH7*), rs12113394 (*LOC100128317*), rs7838015 (*PTDSS1*/*LOC102724804*), rs1053878 (*ABO*), rs8015097 (*KCNH5*), rs62406978 (*RASGEF1C*); CA19-9: rs67300776 (*DPP10*), rs1482587 (*ADAMTS9*-*AS2*/*MAGI1*), rs147309725 (*ADRA1B*/*TTC1*), rs4716999 (*LOC389602*/*LOC285889*); CEA: rs11060471 (*TMEM132D*), rs17568726 (*FARP1*); f-PSA: rs72641143 (*PDE4B*), rs466235 (*PAM*), rs78255505 (*PCAT5*/*ANKRD30A*), rs113984953 (*PABPN1L*/*CBFA2T3*), rs4816598 (*ERG*); SCC-Ag: rs111241781 (*MIR6072*/*LINC00701*), rs117089993 (*HNF4G*/*LINC01111*), rs72773580 (*SHISA9*/*ERCC4*).levelThe specific information was summarized in Table 2. We constructed Manhattan (Fig. 1) and Quantile–quantile (Supplementary Figure 1) plots. The horizontal line in Fig. 1 represents a cut-off value with suggestively genome-wide significance (*p* <5E-6). Supplementary Figure 2 showed a map of the regions associated with each serum tumor marker on different chromosomes.

**Table 2:** Genetic loci significantly associated with tumor markers (8 indicators) identified by the GWAS after imputation analysis in the Han population from Southern China

Tumor maker	Gene	SNP ID	Chr	Position	Alleles	MA	MAF	Func	β	SE	***p***
AFP	LOC100287704/LOC100287834	rs1524622	7	63375308	C/T	C	0.163	intronic	0.424	0.090	3.59E-06
	SGCZ	rs143687479	8	14561503	C/T	C	0.004	intronic	2.585	0.552	3.86E-06
	ZNF444/GALP	rs10425983	19	56167810	A/G	A	0.006	intergenic	-1.995	0.430	4.67E-06
CA50	RBFOX2/APOL3	rs112401204	22	36114041	T/C	T	0.073	intergenic	-0.750	0.150	1.12E-06
	LINC00533/LINC01623	rs9295778	6	28716406	T/G	T	0.062	intergenic	0.782	0.157	1.15E-06
CA125	**GCFC2/LRRTM4**	**rs75332508**	**2**	**76156698**	**T/C**	**T**	**0.013**	**intergenic**	-1.545	0.297	**3.10E-07**
	LINC01790/SLC39A10	rs12476806	2	195329086	C/T	C	0.175	intergenic	0.396	0.084	3.25E-06
	D2HGDH/GAL3ST2	rs7563672	2	241773455	T/G	T	0.358	intergenic	-0.306	0.065	3.70E-06
	LOC728613/MIR4277	rs6554915	5	1639786	C/T	C	0.129	intergenic	0.487	0.103	2.88E-06
	CDK17/CFAP54	rs2028385	12	96423644	G/A	G	0.289	intergenic	0.357	0.071	6.67E-07
CA153	**MUC1**	**rs4072037**	**1**	**155192276**	**C/T**	**C**	**0.161**	**exonic**	1.097	0.110	**1.28E-18**
	PCDH7	rs112804057	4	30878623	G/C	G	0.021	intronic	1.651	0.339	2.61E-06
	**LOC100128317**	**rs12113394**	**7**	**81597863**	**G/A**	**G**	**0.197**	**ncRNA_intronic**	0.692	0.126	**1.45E-07**
	PTDSS1/LOC102724804	rs7838015	8	96340364	C/T	C	0.140	intergenic	0.699	0.144	2.86E-06
	ABO	rs1053878	9	133256264	A/G	A	0.164	exonic	-0.646	0.129	1.33E-06
	KCNH5	rs8015097	14	62895145	G/C	G	0.321	intronic	-0.473	0.098	3.44E-06
	RASGEF1C	rs62406978	5	180204890	T/A	T	0.016	intronic	1.895	0.397	4.12E-06
CA19-9	DPP10	rs67300776	2	115190571	T/G	T	0.347	intronic	-0.322	0.069	4.41E-06
	ADAMTS9-AS2/MAGI1	rs1482587	3	65151572	C/T	C	0.491	intergenic	0.327	0.066	9.00E-07
	ADRA1B/TTC1	rs147309725	5	160000916	A/G	A	0.097	intergenic	-0.543	0.115	3.05E-06
	LOC389602/LOC285889	rs4716999	7	156160296	C/T	C	0.382	intergenic	-0.360	0.071	6.50E-07
CEA	TMEM132D	rs11060471	12	129627766	T/C	T	0.326	intronic	-0.349	0.071	1.25E-06
	FARP1	rs17568726	13	98361516	C/G	C	0.310	intronic	-0.352	0.073	1.99E-06
f-PSA	PDE4B	rs72641143	1	66233311	C/T	C	0.081	intronic	-0.798	0.170	4.78E-06
	PAM	rs466235	5	102762878	C/T	C	0.443	intronic	-0.453	0.095	3.06E-06
	PCAT5/ANKRD30A	rs78255505	10	37091392	C/T	C	0.063	intergenic	-0.892	0.185	2.67E-06
	PABPN1L/CBFA2T3	rs113984953	16	88872490	A/G	A	0.128	intergenic	-0.641	0.134	2.96E-06
	ERG	rs4816598	21	38551999	T/C	T	0.167	intronic	-0.614	0.123	1.22E-06
SCC-Ag	MIR6072/LINC00701	rs111241781	10	2077191	C/T	C	0.035	intergenic	0.917	0.187	1.37E-06
	HNF4G/LINC01111	rs117089993	8	75885175	T/A	T	0.025	intergenic	0.991	0.212	4.10E-06
	SHISA9/ERCC4	rs72773580	16	13562027	A/G	A	0.040	intergenic	-0.878	0.173	6.24E-07

*AFP* Alpha-fetoprotein, *CA50* Carbohydrate Antigen CA-50, *CA125* Carbohydrate Antigen CA125, *CA153* Carbohydrate Antigen CA153, *CA19-9* Carbohydrate Antigen CA19-9, *CEA* Carcino embryonic Antigen, *f-PSA* free Prostate Specific Antigen, *SCC-Ag* Squamous Cell Carcinoma Antigen, *MA* Minor allele, *MAF* Minor allele frequency

*p*-value indicates the level of significant association

*p* < 5E-6 indicates that the candidate SNPs have genome-wide significance

The bold text represents the ‘Top SNPs’ selected for the replication verification experiment (*p* < 5E-7)

### Repeat verification

**Fig. 2:**
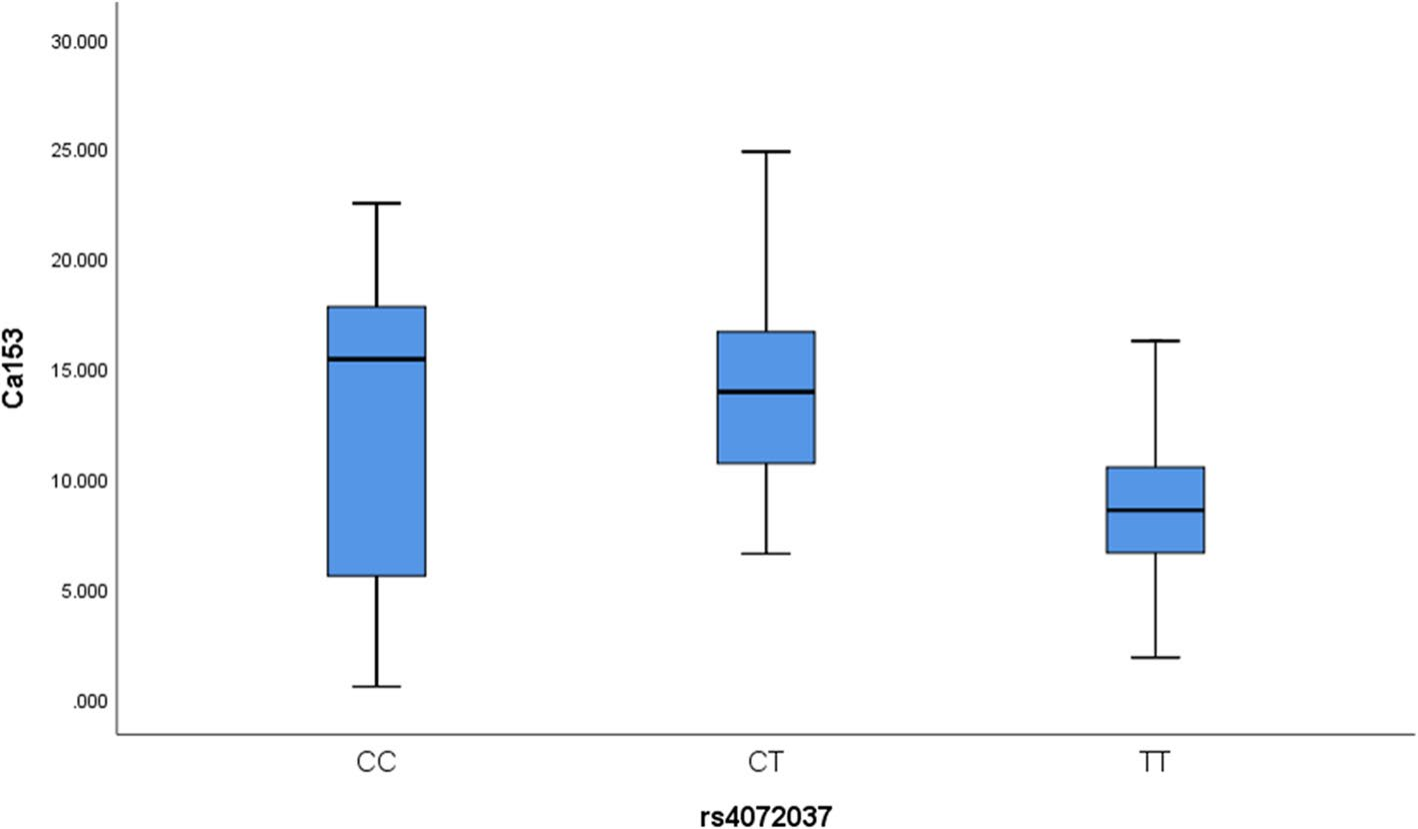
The box plot of CA153 level under different genotypes of rs4072037

We selected TOP SNPs (*p* < 5E-7) from the 31 SNPs identified by GWAS to narrow the scope of verification. Finally, 3 SNPs were selected for the verification test. The results showed (Table 3) that *MUC1*-rs4072037 was still significantly associated with CA153 in different participants (*p* = 3.73E-08, FDR = 2.31E-06). The average level of CA153 under different genotypes of rs4072037 is summarized in Table 4. Compared with the wild genotype TT of *MUC1*-rs4072037, the level of CA153 under the CC/CT genotype was significantly increased (*p* < 0.001). Fig. 2 showed a box plot of the CA153 level changes under different genotypes of rs4072037.

**Table 3:** According to the results of GWAS, SNPs with *p*-value < 5E-07 were selected for replication testing in another population

Tumor maker	Gene	SNP ID	Chr	Position	Alleles	MA	MAF	Func	β	SE	***p***	FDR
CA125	GCFC2; LRRTM4	rs75332508	2	76156698	T/C	T	0.041	intergenic	0.17	0.14	0.070	-
CA153	MUC1	rs4072037	1	155192276	C/T	C	0.176	exonic	-2.78	0.92	3.73E-08*	2.31E-06*
	LOC100128317	rs12113394	7	81597863	G/A	G	0.226	ncRNA_intronic	-2.24	0.95	0.776	-

*CA125* Carbohydrate Antigen CA125, *CA153* Carbohydrate Antigen CA153, *MA* Minor allele, *MAF* Minor allele frequency, *FDR* False Discovery Rate

‘-’ indicates the insignificant results

*p*-value indicates the level of significant association.

*p* < 0.05 indicates that candidate SNP is significantly associated with the tumor maker in participants

**Table 4:** The average concentration of CA153 under different genotypes of rs4072037

Tumor maker	SNP ID	Genotype	N	Mean (U/ml)	Std. Deviation	***p***
**CA153**	**rs4072037**	CC	8 (2.0%)	12.57	8.19	< 0.001*
		CT	124 (31.2%)	13 .47	5.42	
		TT	266 (66.8%)	8.66	3.72	
		Total	398 (100%)	10.24	4.96	

The number of participants (N)

*p* < 0.05 indicates that the concentration of the tumor marker is significantly different under different genotypes

## Discussion

GWAS results of our study showed that only *MUC1*-rs4072037 significantly genome-wide associated with CA153 (*p* = 1.28E-18). However, a total of 30 genetic loci were identified in the Han population from Southern China, which were suggestively genome-wide associated with level of TMs. In addition, the results of repeated verification showed that only *MUC1* rs4072037 still had a significant association with CA153 level.

The level of CA15-3 is increased in the serum of patients with malignant breast cancer and ovarian cancer [23, 24]. In this study, we found that the association between rs4072037 (*MUC1*) and CA15-3 levels reached the whole genome level. In addition, there were seven genetic loci that have a suggestive genome-wide significant association with CA153 level. Among the genetic loci identified in this study, rs4072037 (*MUC1*) [25, 26] and rs1053878 (*ABO*) [27] have been reported to be associated with the occurrence and development of cancers. However, their specific mechanism in cancer remains unclear. Gu, X. et al. found the important evidence that *MUC1* rs4072037 may be used as a tumor marker in a large sample of ‘case Vs. control’ study [28]. Combining the results of this study, we speculate that *MUC1*-rs4072037 and *ABO*-rs1053878 may affect the expression level of CA153, thereby affecting the occurrence and development of cancer.

We only conducted GWAS of CA153 among 165 participants due to data missing in sample collection. Nevertheless, MUC1 rs4072037 was still detected to be significantly associated with CA153 levels during the repeated verification, which was passed FDR correction. It shows that this study result is worth believing. And compared with the TT genotype, the CA153 level under the CC or CT genotype was significantly increased. Recent studies have shown that the median expression of CA153 is significantly higher in a variety of cancers (lung cancer, breast cancer, ovarian cancer, cervical cancer) [29]. Combined with the results of our study, the appearance of the minor allele ‘C’ will significantly increase the level of CA153. Based on the above, *MUC1* rs4072037 can be used as a new genetic signal for tumor prevention in southern Chinese Han population. To our knowledge, our study is the first to report a significant association between *MUC1* rs4072037 and CA153 level. Our study will provide a valuable reference for tumor prevention.

The median survival rate of hepatocellular carcinoma patients with high serum AFP level is low [30]. A Japanese GWAS study found that the AT/TT genotype of rs17047200 on the *TLL1* intron was associated with the e level of AFP [31]. Perhaps due to the different study methods, sample size or the genetic backgrounds of the participating populations, our results are different from the previous studies. In our study, the GWAS results showed that the genetic loci that have genome-wide significance with the AFP level in the Han population of southern China are rs1524622 (LOC100287704/LOC100287834), rs143687479 (*SGCZ*), rs10425983 (*ZNF444*/*GALP*). As far as we know, these three SNPs were discovered for the first time that may be associated with changes in AFP level.

High CEA level are associated with tumorigenesis and are often used to assist in the diagnosis of cancers caused by the gastrointestinal tract [32]. Carcinoembryonic antigen (CEA) is also highly expressed in malignant breast cancer [23]. Up to now, there were studies have identified 5 genetic variants of the ABO gene associated with CEA level through GWAS [13]. However, the association between the two SNPs (*TMEM132D*-rs11060471; *FARP1*-rs17568726) identified by GWAS and CEA level changes in our study was reported for the first time. They may be new genetic signals to monitor changes of CEA level in cancer patients, but this requires further experiments to verify.

Serum f-PSA is an important indicator for early detection of prostate cancer. Jeannette et al. found that KLK3 SNPs may be associated with f-PSA level in the GWAS assessment conducted among African Americans and Europeans [11]. Our results are different from those of previous studies. We found that the SNPs of *PDE4B*, *PAM*, *PCAT5*/*ANKRD30A*, *PABPN1L*/*CBFA2T3* were significantly associated with changes in f-PSA level among the Chinese Southern Han population. We speculate that the reason for this difference may be influenced by different genetic background or environment. Nevertheless, our results still provide data supplements for GWAS studies on f-PSA in different populations.

Detection of serum SCC-Ag level can be used in the early diagnosis of cervical cancer recurrence [33]. Up to now, there is no study on the SCC-Ag GWAS has been reported. We conducted the SCC-Ag GWAS study for the first time in the Han population of southern China. And three genetic loci (rs111241781, rs117089993, rs72773580) have been identified that may be associated with the SCC-Ag level. They may be used as new genetic signals for early diagnosis of cervical cancer recurrence.

Carbohydrate antigen (CA) plays a key role in tumor progression, CA15-3, CA125, and CA19-9 are widely used in various cancer screening [34]. Studies have reported that CA19-9 is the most sensitive tumor marker for pancreatic cancer [3]. And the expression of CA19-9 is related to the recurrence [35]. CA125 is a reliable marker for clinical diagnosis of ovarian cancer [7], and its increasing level is a signal of recurrence of female germline tumors [36]. The expression of CA50 in the serum of pancreatic cancer patients was significantly higher than that of healthy controls [9]. Although some SNPs that are significantly associated to the level of CA19-9/CA125 have been reported in previous GWAS studies [13, 37], the genetic loci identified in our study have never been reported in Southern Chinese Han population. Our results provide reliable evidence that these SNPs can be used as new genetic signals for one or some tumor markers.

The characteristics and changes of tumor markers has important guiding significance for health assessment management, cancer prevention and tumor detection. Our study provides important evidence for in-depth understanding of the mechanism of serum tumor markers. The small sample size of this study is a limitation that cannot be ignored. In subsequent studies, we will expand the sample size or replace populations with different genetic backgrounds as study subjects to conduct validation tests. In any case, our study have provided a valuable reference for clinical monitoring and diagnosis of the occurrence and development of tumors among Han population from southern China.

## Conclusion

In summary, *MUC1*-rs4072037 is significantly genome-wide associated with CA153 level (*p* = 1.28E-18) and 30 genetic loci were suggestively genome-wide associated with level of tumor markers (AFP, CA50, CA125, CA153, CA19-9, CEA, f-PSA, SCC-Ag) among the Han population from Southern China. Our study provides a valuable reference for clinical tumor prevention, and also provides a new theoretical basis for the phenotype research of tumor markers in healthy populations and individual health management.

## Supplementary Information

**Additional file 1: Supplemental Figure 1.** Quantile–quantile plots about the results of the GWAS. (A) AFP; (B) CA50; (C) CA125; (D) CA153; (E) CA19-9; (F) CEA; (G) f-PSA; (H) SCC-Ag.

**Additional file 2: Supplemental Figure 2.** A map of the SNPs associated with each serum tumor marker on different chromosomes. (A) AFP; (B) CA50; (C) CA125; (D) CA153; (E) CA19-9; (F) CEA; (G) f-PSA; (H) SCC-Ag.

## Data Availability

The datasets used and analyzed in the current study are available from the corresponding author on reasonable request.

## Abbreviations

TMs: Tumor markers
AFP: Alpha fetoprotein
CEA: Carcino embryonic antigen
f-PSA: Free Prostate Specific Antigen
SCC-Ag: Squamous cell carcinoma antigen
GWAS: Genome-wide association study

## References

[1] Cai J, Chen L (2019). Genome-wide mapping of 5-hydroxymethylcytosines in circulating cell-free DNA as a non-invasive approach for early detection of hepatocellular carcinoma. Gut.

[2] Chen W, Peng J, Ye J, Dai W, Li G, He Y (2020). Aberrant AFP expression characterizes a subset of hepatocellular carcinoma with distinct gene expression patterns and inferior prognosis. J Cancer.

[3] Scarà S, Bottoni P, Scatena R (2015). CA 19-9: Biochemical and Clinical Aspects. Adv Exp Med Biol.

[4] Yajima H, Omura N, Matai K, Mitsumori N, Yoshida K, Yanaga K (2014). Clinicopathological features of CA19-9-producing gastric cancer. Hepatogastroenterology..

[5] Vanderbeck K, Voutsadakis IA (2016). Elevation of Ca19-9 tumor antigen in colorectal cancer: an in silico investigation of pathogenesis. Int J Color Dis.

[6] Chen Z, Liu Z (2020). Combination of CA19-9 and the neutrophil-to-lymphocyte ratio for the differential diagnosis of gallbladder carcinoma. Cancer Manag Res.

[7] Felder M, Kapur A, Gonzalez-Bosquet J, Horibata S, Heintz J, Albrecht R (2014). MUC16 (CA125): tumor biomarker to cancer therapy, a work in progress. Mol Cancer.

[8] Bon GG, Kenemans P, Verstraeten AA, Go S, Philipi PA, van Kamp GJ (2001). Maternal serum Ca125 and Ca15-3 antigen levels in normal and pathological pregnancy. Fetal Diagn Ther.

[9] Lei XF, Jia SZ, Ye J, Qiao YL, Zhao GM, Li XH (2017). Application values of detection of serum CA199, CA242 and CA50 in the diagnosis of pancreatic cancer. J Biol Regul Homeost Agents.

[10] Bajenova O, Gorbunova A, Evsyukov I, Rayko M, Gapon S, Bozhokina E (2016). The genome-wide analysis of carcinoembryonic antigen signaling by colorectal cancer cells using RNA sequencing. PLoS One.

[11] Bensen JT, Xu Z, Smith GJ, Mohler JL, Fontham ET, Taylor JA (2013). Genetic polymorphism and prostate cancer aggressiveness: a case-only study of 1,536 GWAS and candidate SNPs in African-Americans and European-Americans. Prostate..

[12] Hamada K, Hanakawa Y, Hashimoto K, Iwamoto M, Kihana T, Hirose S (2001). Gene expression of human squamous cell carcinoma antigens 1 and 2 in human cell lines. Oncol Rep.

[13] He M, Wu C, Xu J, Guo H, Yang H, Zhang X (2014). A genome wide association study of genetic loci that influence tumour biomarkers cancer antigen 19-9, carcinoembryonic antigen and α fetoprotein and their associations with cancer risk. Gut..

[14] Zhang G, Xu Y, Lu X, Huang H, Zhou Y, Lu B (2009). Diagnosis value of serum B7-H3 expression in non-small cell lung cancer. Lung Cancer.

[15] Evdokimova VN, Liu Y, Potter DM, Butterfield LH (2007). AFP-specific CD4+ helper T-cell responses in healthy donors and HCC patients. J Immunother.

[16] Hatate K, Yamashita K, Hirai K, Kumamoto H, Sato T, Ozawa H (2008). Liver metastasis of colorectal cancer by protein-tyrosine phosphatase type 4A, 3 (PRL-3) is mediated through lymph node metastasis and elevated serum tumor markers such as CEA and CA19-9. Oncol Rep.

[17] Motzer RJ, Hutson TE, Hudes GR, Figlin RA, Martini JF, English PA (2014). Investigation of novel circulating proteins, germ line single-nucleotide polymorphisms, and molecular tumor markers as potential efficacy biomarkers of first-line sunitinib therapy for advanced renal cell carcinoma. Cancer Chemother Pharmacol.

[18] Hirschhorn JN, Daly MJ (2005). Genome-wide association studies for common diseases and complex traits. Nat Rev Genet.

[19] Berković MC, Jokić M, Marout J, Radosević S, Zjacić-Rotkvić V, Kapitanović S (2010). IL-2 -330 T/G SNP and serum values-potential new tumor markers in neuroendocrine tumors of the gastrointestinal tract and pancreas (GEP-NETs). J Mol Med (Berl).

[20] Zhang J, Chi H, Xiao H, Tian X, Wang Y, Yun X (2017). Interleukin 6 (IL-6) and Tumor Necrosis Factor α (TNF-α) Single Nucleotide Polymorphisms (SNPs), Inflammation and Metabolism in Gestational Diabetes Mellitus in Inner Mongolia. Med Sci Monit.

[21] Grigorova AA, Trenova AG, Stanilova SA (2021). Association of polymorphism -308G/A in tumor necrosis factor-alpha gene (TNF-α) and TNF-α serum levels in patients with relapsing-remitting multiple sclerosis. Neurol Res.

[22] Son HY, Hwangbo Y, Yoo SK, Im SW, Yang SD, Kwak SJ (2017). Genome-wide association and expression quantitative trait loci studies identify multiple susceptibility loci for thyroid cancer. Nat Commun.

[23] Dolscheid-Pommerich RC, Keyver-Paik M, Hecking T, Kuhn W, Hartmann G, Stoffel-Wagner B (2017). Clinical performance of LOCI™-based tumor marker assays for tumor markers CA 15-3, CA 125, CEA, CA 19-9 and AFP in gynecological cancers. Tumour Biol.

[24] Williams KA, Terry KL, Tworoger SS, Vitonis AF, Titus LJ, Cramer DW (2014). Polymorphisms of MUC16 (CA125) and MUC1 (CA15.3) in relation to ovarian cancer risk and survival. PLoS One.

[25] Duan F, Song C, Dai L, Cui S, Zhang X, Zhao X (2014). The effect of MUC1 rs4072037 functional polymorphism on cancer susceptibility: evidence from published studies. PLoS One.

[26] Kim BS, Lee I (2020). Association between the MUC1 rs4072037 Polymorphism and Risk of Gastric Cancer and. Clin Outcomes.

[27] Cozzi GD, Levinson RT, Toole H, Snyder MR, Deng A, Crispens MA (2017). Blood type, ABO genetic variants, and ovarian cancer survival. PLoS One.

[28] Gu X, Feng J, Liu L, Lu M, Ma X, Cao Y (2018). Association of MUC1 rs4072037 Functional Polymorphism and Cancer Risk: Evidence from 12551 Cases and 13436 Controls. J Cancer.

[29] Wang Y, Lei L, Chi YG, Liu LB, Yang BP (2019). A comprehensive understanding of ovarian carcinoma survival prognosis by novel biomarkers. Eur Rev Med Pharmacol Sci.

[30] Tangkijvanich P, Anukulkarnkusol N, Suwangool P, Lertmaharit S, Hanvivatvong O, Kullavanijaya P (2000). Clinical characteristics and prognosis of hepatocellular carcinoma: analysis based on serum alpha-fetoprotein levels. J Clin Gastroenterol.

[31] Matsuura K, Sawai H, Ikeo K, Ogawa S, Iio E, Isogawa M (2017). Genome-Wide Association Study Identifies TLL1 Variant Associated With Development of Hepatocellular Carcinoma After Eradication of Hepatitis C Virus Infection. Gastroenterology..

[32] Goldenberg DM, Neville AM, Carter AC, Go VL, Holyoke ED, Isselbacher KJ (1981). CEA (carcinoembryonic antigen): its role as a marker in the management of cancer. J Cancer Res Clin Oncol.

[33] Salvatici M, Achilarre MT, Sandri MT, Boveri S, Vanna Z, Landoni F (2016). Squamous cell carcinoma antigen (SCC-Ag) during follow-up of cervical cancer patients: Role in the early diagnosis of recurrence. Gynecol Oncol.

[34] Sturgeon C (2002). Practice guidelines for tumor marker use in the clinic. Clin Chem.

[35] Sakamoto T, Saito H, Uchinaka EI, Morimoto M, Amisaki M, Tokuyasu N (2018). The Combination of Neutrophil-to-lymphocyte Ratio and Serum Carbohydrate Antigen 19-9 Level as a Prognostic Indicator in Patients with Recurrent Pancreatic Cancer. Anticancer Res.

[36] Xiao C, Zhao J, Guo P, Wang D, Zhao D, Ren T (2014). Clinical analysis of primary primitive neuroectodermal tumors in the female genital tract. Int J Gynecol Cancer.

[37] Rupp C, Friedrich K, Folseraas T, Wannhoff A, Bode KA, Weiss KH (2014). Fut2 genotype is a risk factor for dominant stenosis and biliary candida infections in primary sclerosing cholangitis. Aliment Pharmacol Ther.

