# Supplementary figures and images for: Genome-wide association study of serum tumor markers in Southern Chinese Han population
Source: BMC Cancer. 2022 Feb 10;22:160. doi: 10.1186/s12885-022-09236-6 (PMC8832811; doi:10.1186/s12885-022-09236-6)

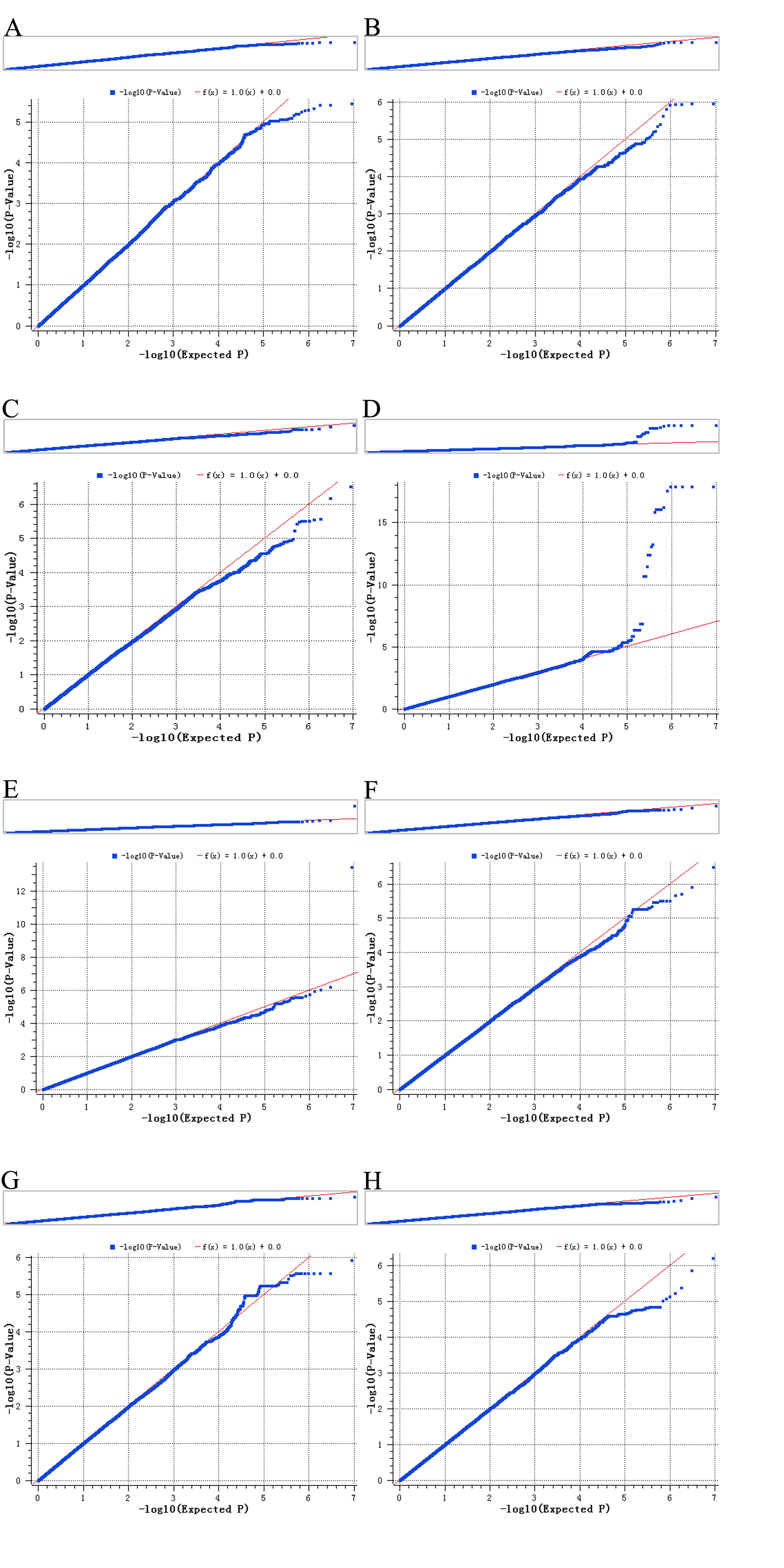

Supplement: Supplementary file 1 — Additional file 1: Supplemental Figure 1. Quantile–quantile plots about the results of the GWAS. (A) AFP; (B) CA50; (C) CA125; (D) CA153; (E) CA19-9; (F) CEA; (G) f-PSA; (H) SCC-Ag. [file 12885_2022_9236_MOESM1_ESM.tif]

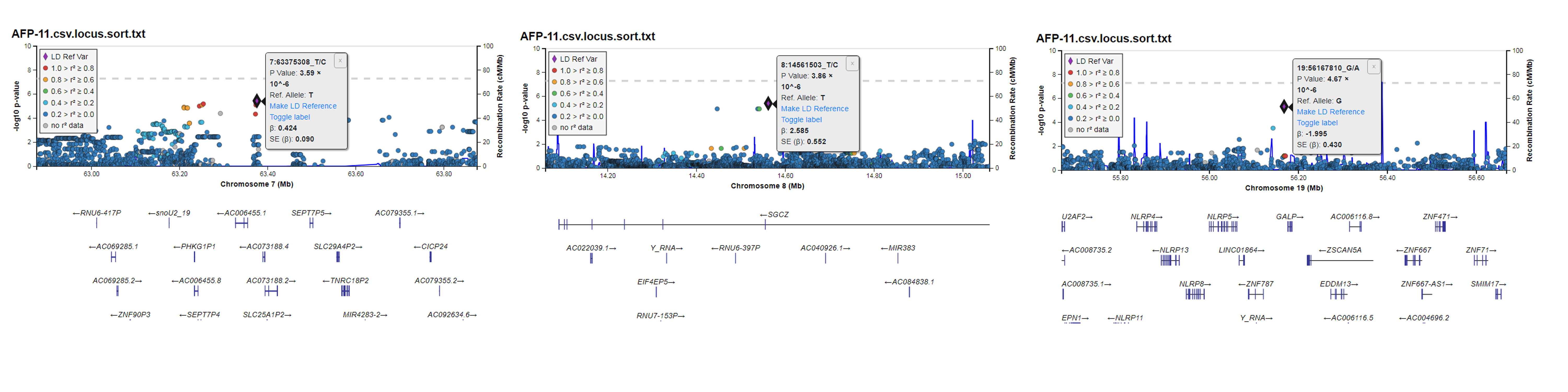

Supplement: Supplementary file 2 — Additional file 2: Supplemental Figure 2. A map of the SNPs associated with each serum tumor marker on different chromosomes. (A) AFP; (B) CA50; (C) CA125; (D) CA153; (E) CA19-9; (F) CEA; (G) f-PSA; (H) SCC-Ag. [file 12885_2022_9236_MOESM2_ESM.zip › Supplemental figure 2A.tif]

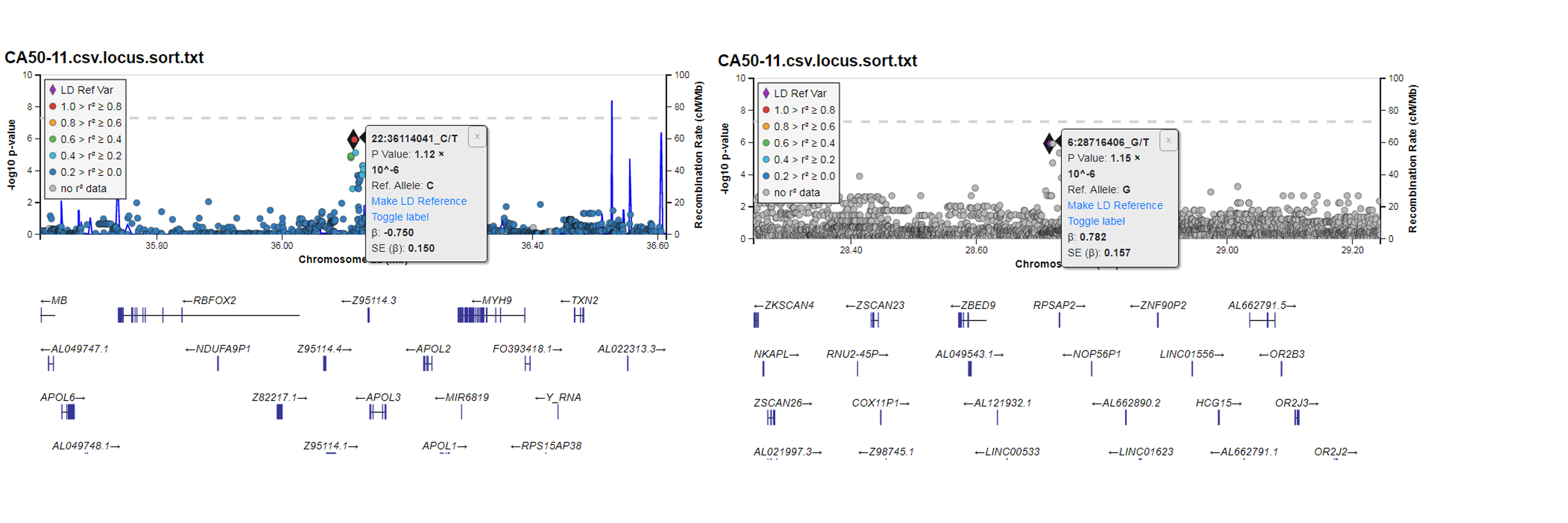

Supplement: Supplementary file 2 — Additional file 2: Supplemental Figure 2. A map of the SNPs associated with each serum tumor marker on different chromosomes. (A) AFP; (B) CA50; (C) CA125; (D) CA153; (E) CA19-9; (F) CEA; (G) f-PSA; (H) SCC-Ag. [file 12885_2022_9236_MOESM2_ESM.zip › Supplemental figure 2B.tif]

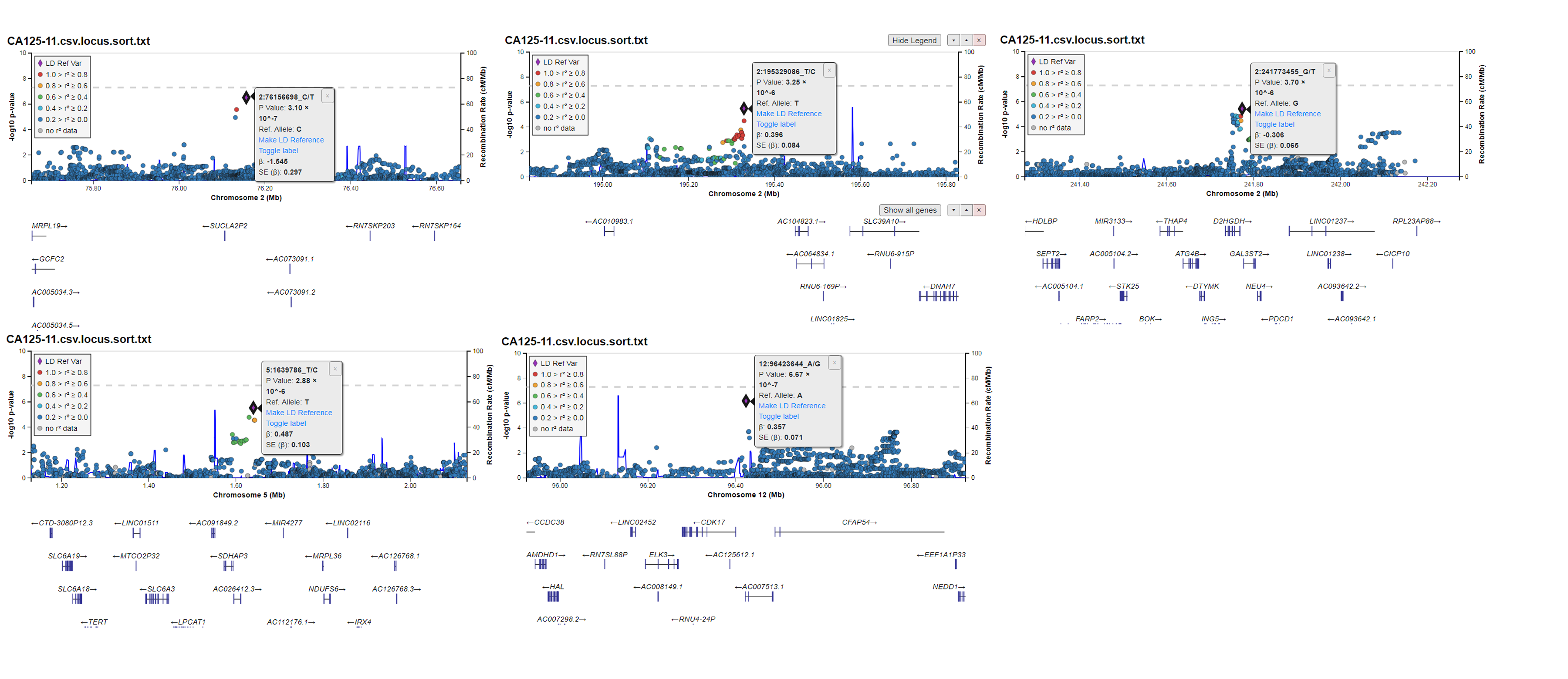

Supplement: Supplementary file 2 — Additional file 2: Supplemental Figure 2. A map of the SNPs associated with each serum tumor marker on different chromosomes. (A) AFP; (B) CA50; (C) CA125; (D) CA153; (E) CA19-9; (F) CEA; (G) f-PSA; (H) SCC-Ag. [file 12885_2022_9236_MOESM2_ESM.zip › Supplemental figure 2C.tif]

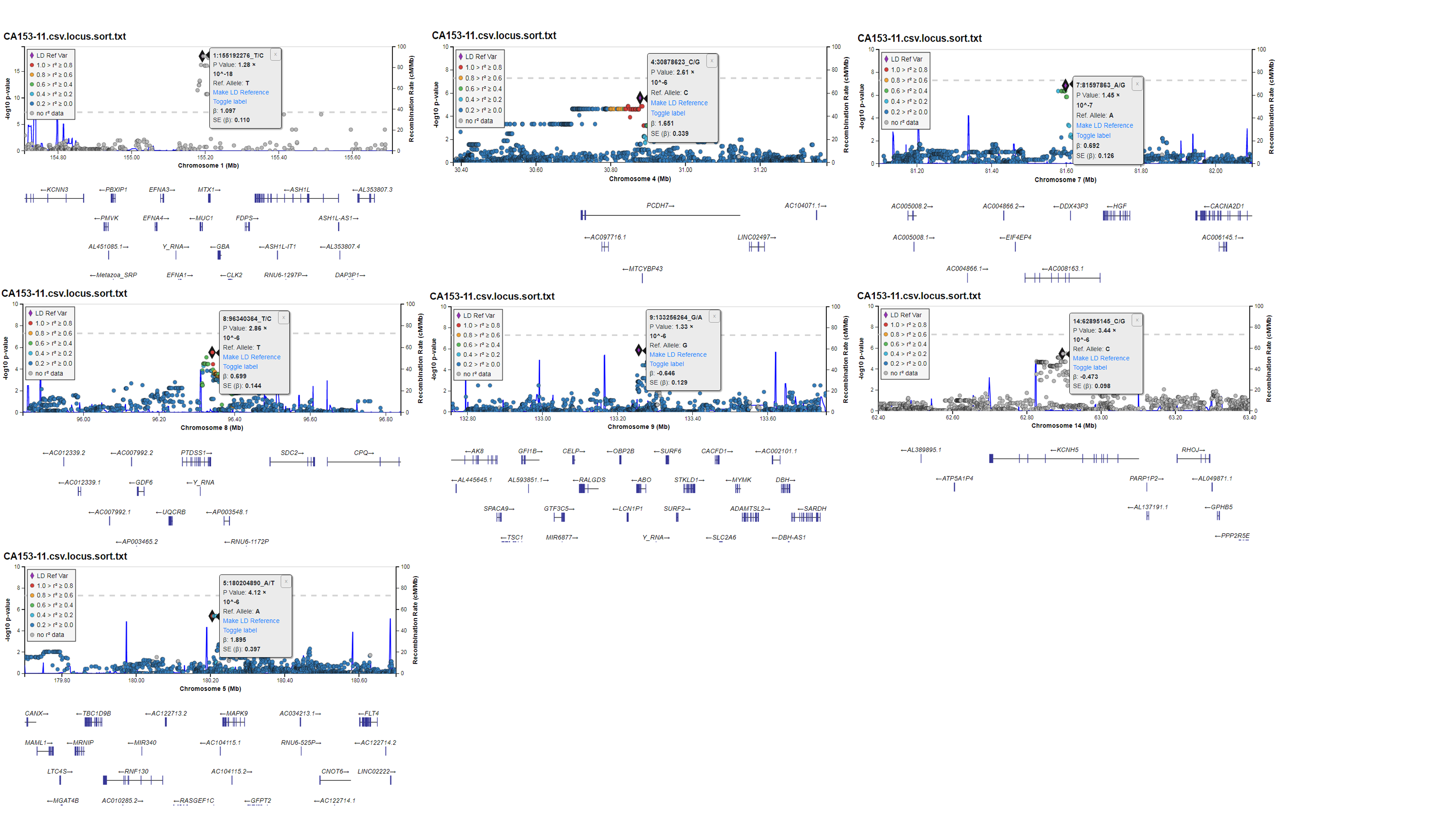

Supplement: Supplementary file 2 — Additional file 2: Supplemental Figure 2. A map of the SNPs associated with each serum tumor marker on different chromosomes. (A) AFP; (B) CA50; (C) CA125; (D) CA153; (E) CA19-9; (F) CEA; (G) f-PSA; (H) SCC-Ag. [file 12885_2022_9236_MOESM2_ESM.zip › Supplemental figure 2D.tif]

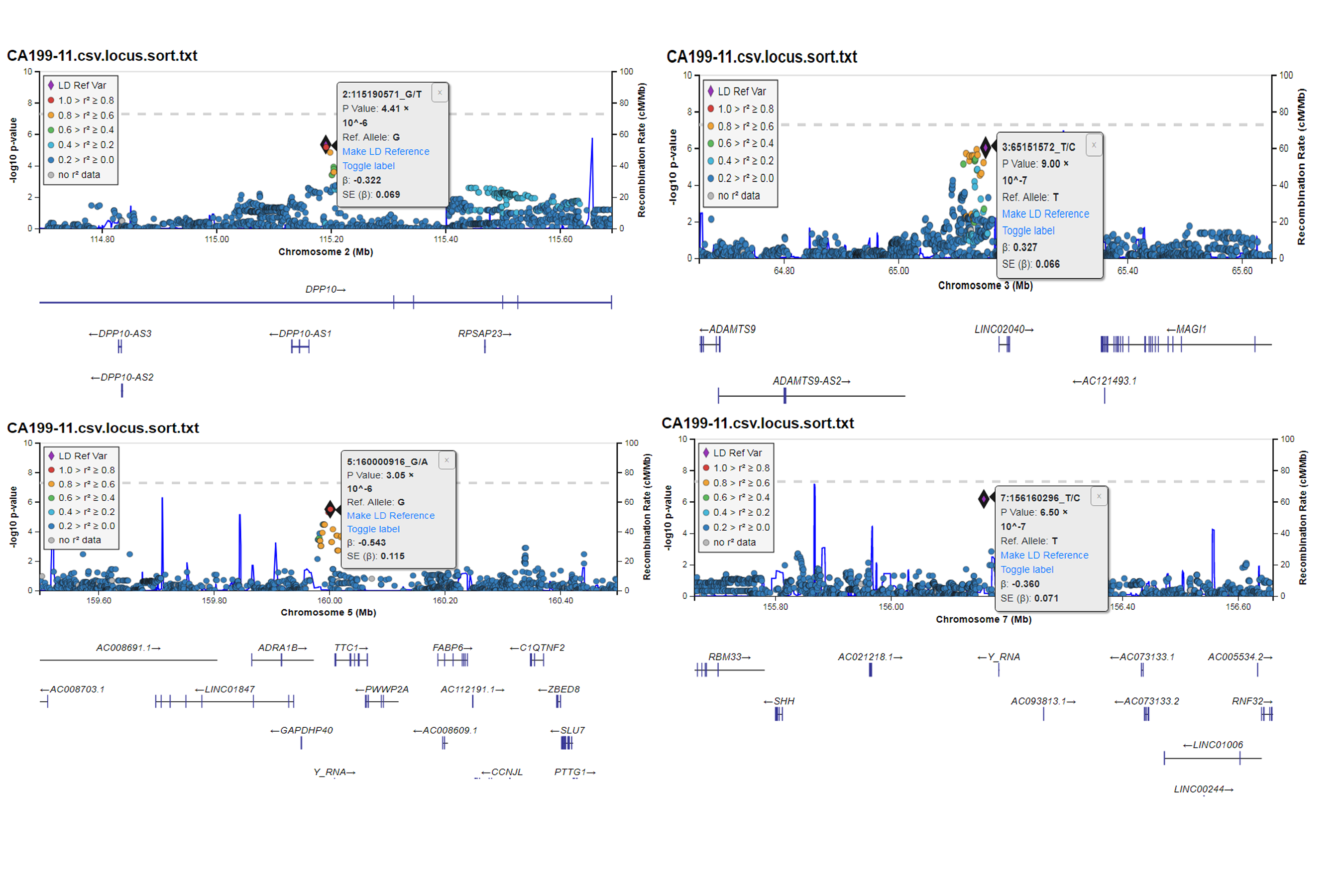

Supplement: Supplementary file 2 — Additional file 2: Supplemental Figure 2. A map of the SNPs associated with each serum tumor marker on different chromosomes. (A) AFP; (B) CA50; (C) CA125; (D) CA153; (E) CA19-9; (F) CEA; (G) f-PSA; (H) SCC-Ag. [file 12885_2022_9236_MOESM2_ESM.zip › Supplemental figure 2E.tif]

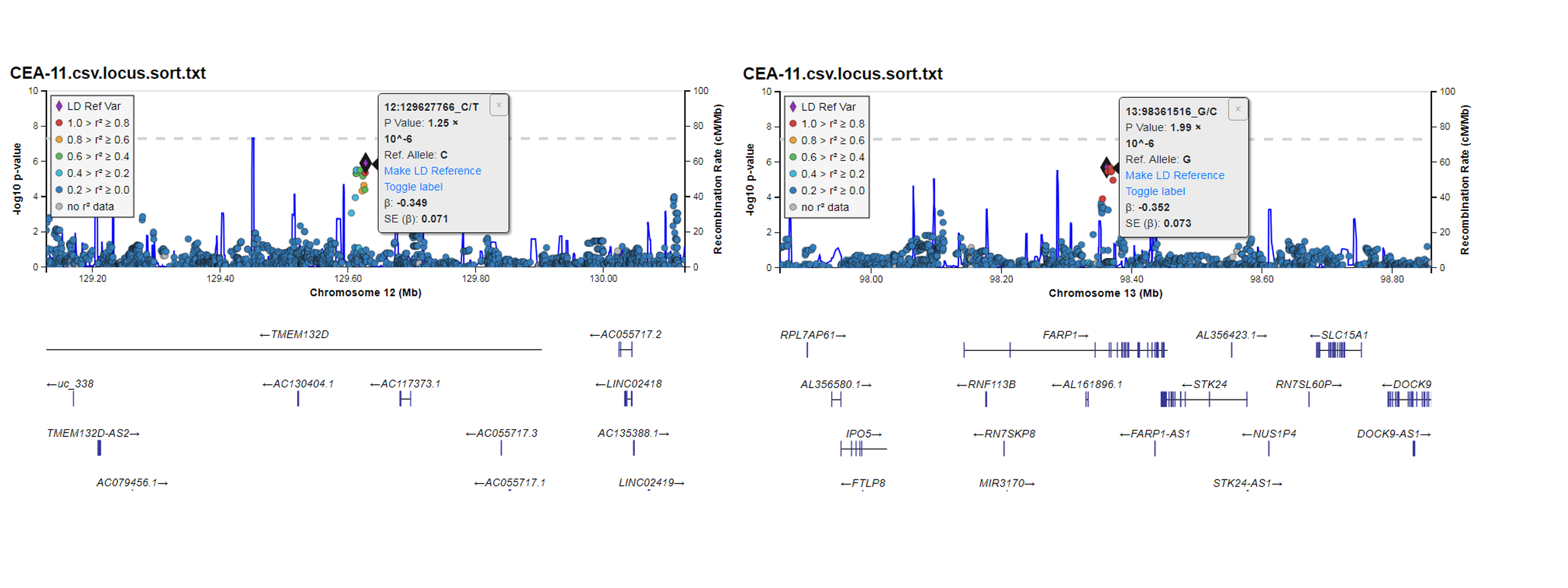

Supplement: Supplementary file 2 — Additional file 2: Supplemental Figure 2. A map of the SNPs associated with each serum tumor marker on different chromosomes. (A) AFP; (B) CA50; (C) CA125; (D) CA153; (E) CA19-9; (F) CEA; (G) f-PSA; (H) SCC-Ag. [file 12885_2022_9236_MOESM2_ESM.zip › Supplemental figure 2F.tif]

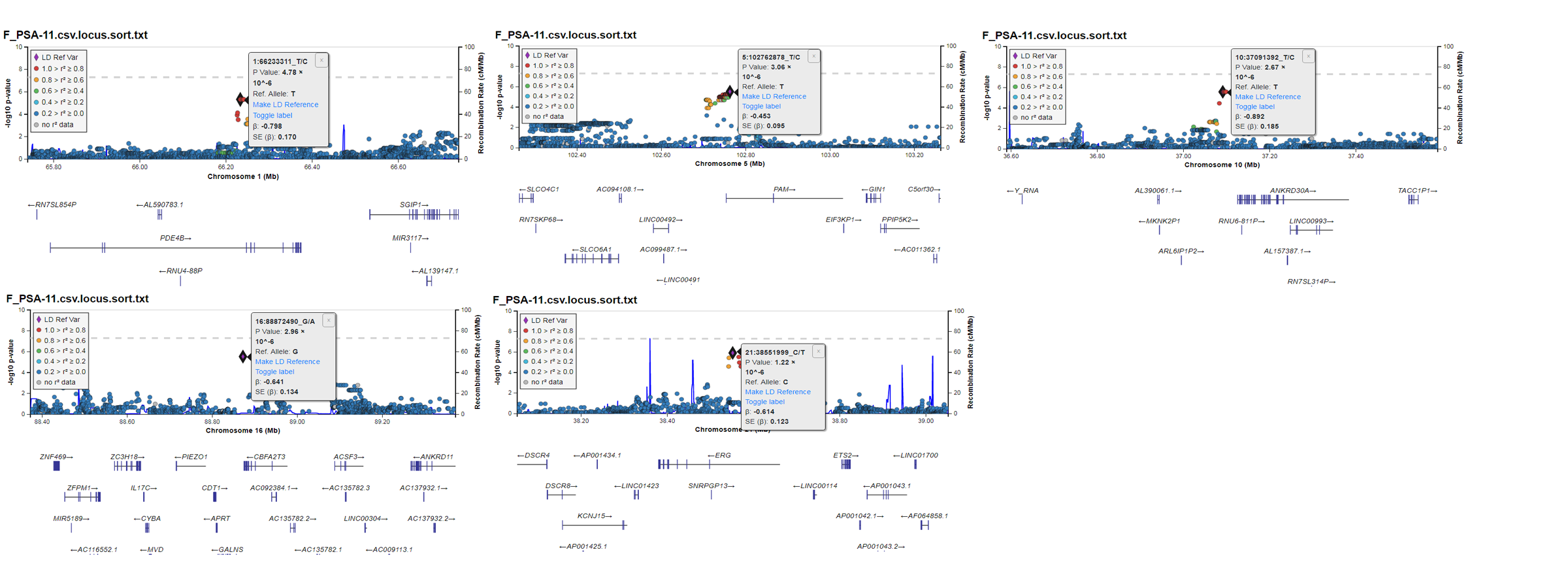

Supplement: Supplementary file 2 — Additional file 2: Supplemental Figure 2. A map of the SNPs associated with each serum tumor marker on different chromosomes. (A) AFP; (B) CA50; (C) CA125; (D) CA153; (E) CA19-9; (F) CEA; (G) f-PSA; (H) SCC-Ag. [file 12885_2022_9236_MOESM2_ESM.zip › Supplemental figure 2G.tif]

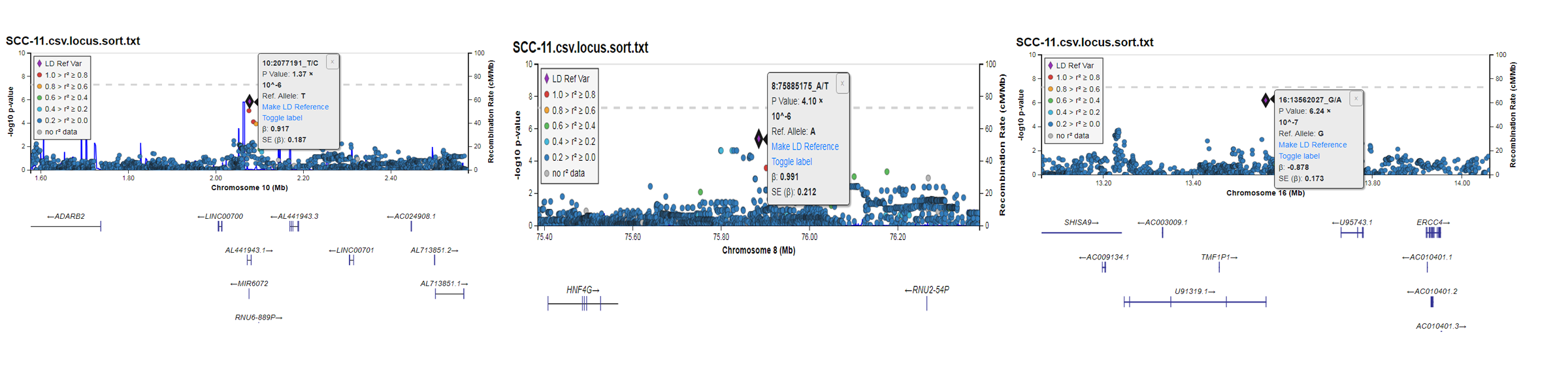

Supplement: Supplementary file 2 — Additional file 2: Supplemental Figure 2. A map of the SNPs associated with each serum tumor marker on different chromosomes. (A) AFP; (B) CA50; (C) CA125; (D) CA153; (E) CA19-9; (F) CEA; (G) f-PSA; (H) SCC-Ag. [file 12885_2022_9236_MOESM2_ESM.zip › Supplemental figure 2H.tif]
